# Genetics of substance use disorders: a review

**DOI:** 10.1017/S0033291721000969

**Published:** 2021-10

**Authors:** Joseph D. Deak, Emma C. Johnson

**Affiliations:** 1 Department of Psychiatry, Yale School of Medicine, New Haven, CT, USA; 2 Department of Psychiatry, Veterans Affairs Connecticut Healthcare Center, West Haven, CT, USA; 3 Department of Psychiatry, Washington University School of Medicine, St. Louis, MO, USA

**Keywords:** substance use disorders, genetics, genome wide association study, twin and family studies, heritability, genetic epidemiology

## Abstract

Substance use disorders (SUDs) are prevalent and result in an array of negative consequences. They are influenced by genetic factors (*h^2^* = ~50%). Recent years have brought substantial progress in our understanding of the genetic etiology of SUDs and related traits. The present review covers the current state of the field for SUD genetics, including the epidemiology and genetic epidemiology of SUDs, findings from the first-generation of SUD genome-wide association studies (GWAS), cautions about translating GWAS findings to clinical settings, and suggested prioritizations for the next wave of SUD genetics efforts. Recent advances in SUD genetics have been facilitated by the assembly of large GWAS samples, and the development of state-of-the-art methods modeling the aggregate effect of genome-wide variation. These advances have confirmed that SUDs are highly polygenic with many variants across the genome conferring risk, the vast majority of which are of small effect. Downstream analyses have enabled finer resolution of the genetic architecture of SUDs and revealed insights into their genetic relationship with other psychiatric disorders. Recent efforts have also prioritized a closer examination of GWAS findings that have suggested non-uniform genetic influences across measures of substance use (e.g. consumption) and problematic use (e.g. SUD). Additional highlights from recent SUD GWAS include the robust confirmation of loci in alcohol metabolizing genes (e.g. *ADH1B* and *ALDH2*) affecting alcohol-related traits, and loci within the *CHRNA5-CHRNA3-CHRNB4* gene cluster influencing nicotine-related traits. Similar successes are expected for cannabis, opioid, and cocaine use disorders as sample sizes approach those assembled for alcohol and nicotine.

## Introduction

Substance use disorders (SUDs) are heritable psychiatric disorders [Diagnostic and Statistical Manual of Mental Disorders, Fifth Edition (DSM-5); American Psychiatric Association, 2013] that are influenced by both environmental and genetic factors. Given the public health burden of SUDs, a better understanding of SUD etiology is of wide-reaching importance. Genetic studies have begun to elucidate the molecular mechanisms underlying SUDs and related traits, including other psychiatric conditions with which SUDs frequently co-occur (Grant et al., 2016; Kessler, 2004). While there are challenges inherent in studying complex, polygenic traits such as SUDs, it is hoped that better understanding the genetic basis of risk for developing SUDs will eventually help inform SUD prevention and treatment. In this review, we cover SUD epidemiology, conclusions from twin and family studies of SUDs, and findings from more recent molecular genetic studies^1^; finally, we summarize the current state of the field and suggest future directions.

## Definition of SUD

SUDs are defined by the DSM-5 (American Psychiatric Association, 2013) as the presence of at least two of 11 criteria in a 12-month period, with disorder severity indexed by the number of criteria endorsed (2–3 = mild; 4–5 = moderate; ⩾6 = severe). Broadly speaking, DSM-5 SUD criteria correspond to the presence of substance-related problems, such as increased use, unsuccessful attempts to stop or cut down, continued use despite negative physical, psychological, and social consequences, persistent craving, development of tolerance, and symptoms of withdrawal. These criteria can be assessed in relation to multiple substances (e.g. alcohol, nicotine, cannabis, opioid, cocaine).

Prior to DSM-5, DSM-IV distinguished substance abuse from dependence. Substance abuse required the endorsement of at least one of four abuse criteria, while substance dependence required at least three of seven dependence criteria. Research favored a unidimensional diagnosis over the separation of abuse and dependence (Hasin et al., 2013). With the revised DSM-5 SUD criteria, the substance abuse ‘legal problems’ criterion was removed and a craving criterion was added. Compton, Dawson, Goldstein, and Grant (2013) found that a threshold of ⩾4 DSM-5 criteria (i.e. moderate severity) demonstrated optimal correspondence with DSM-IV dependence for alcohol, cocaine, and opioid use disorders.

The present review will focus on five SUDs that have demonstrated substantial progress in molecular genetics in recent years: alcohol use disorder (AUD), nicotine use disorder (NicUD), cannabis use disorder (CanUD), opioid use disorder (OUD), and cocaine use disorder (CocUD). Many studies reviewed utilized DSM-IV defined substance abuse and dependence.

## Epidemiology

SUDs are highly prevalent behaviors associated with an array of negative outcomes. Epidemiological estimates suggest that up to 29.1% and 27.9% of individuals will meet the criteria for AUD and NicUD, respectively, in their lifetime, with lower lifetime prevalence rates for CanUD (6.3%), OUD (2.1%), and CocUD (2.4%) (Grant et al., 2016). All SUDs contribute to increased rates of injuries, elevated risk of other disorders, and pose a large economic cost worldwide (Degenhardt & Hall, 2012). For example, AUD and NicUD, respectively, contribute to 3 million (5.3%) and 7 million (12.3%) worldwide deaths annually, making both among the leading causes of global mortality (Global Status Report on Alcohol and Health, 2018; WHO Report on the Global Tobacco Epidemic, 2017). SUDs that occur at lower rates (e.g. OUD) also have severe impact; the USA is currently combating an opioid use public health crisis, with an estimated 47 600 individuals dying from opioid overdoses in 2018 (Hedegaard, Miniño, & Warner, 2020).

As reviewed by Koob and Le Moal (2001), the cycle of addictions (including SUDs) can be thought of as having three main components: preoccupation-anticipation, binge-intoxication, and withdrawal-negative affect. This model aligns with DSM criteria, while allowing for the influence of genetic vulnerabilities and environmental risk and protective factors at different stages. New research also suggests this stage-based model is supported by genomic data (Hatoum et al., 2021). Family history of SUDs, peer substance use, lower socioeconomic status (SES), and psychiatric comorbidities are all associated with increased risk of developing a SUD (Stone, Becker, Huber, & Catalano, 2012). Some of these are potential *consequences* of SUDs as well as risk factors (e.g. socioeconomic hardship, poorer mental health prognosis), and contribute to the negative outcomes associated with SUDs (Kendler, Ohlsson, Karriker-Jaffe, Sundquist, & Sundquist, 2017).

## Genetic epidemiology

Twin and family studies have demonstrated strong familial inheritance patterns for SUDs (Prom-Wormley, Ebejer, Dick, & Bowers, 2017). Heritability (*h*^2^) estimates across SUDs vary, but broadly suggest that genetic influences account for approximately 50% of the risk. Quantitative genetic studies have also suggested that, in addition to the presence of substance-specific influences for SUDs – with nicotine and opiates showing the most evidence of substance-specific genetic factors (Kendler, Myers, & Prescott, 2007; Tsuang et al., 1998) – there are heritable factors that contribute to SUDs more broadly (Kendler et al., 2007; Kendler, Prescott, Myers, & Neale, 2003).

### Alcohol use disorder

Heritability estimates for AUD range from ~0.50 to 0.64 (Heath et al., 1997; Kendler, 2001), with a recent meta-analysis reporting *h*^2^ of ~0.50 (Verhulst, Neale, & Kendler, 2015). Heritability estimates for AUD diagnosis tend to be slightly higher than for other alcohol-related traits, such as alcohol use initiation (*h*^2^ = 0.30–0.40; Koopmans, Slutske, Van Baal, & Boomsma, 1999) and alcohol use frequency (*h*^2^ = 0.37–0.47; Viken, Kaprio, Koskenvuo, & Rose, 1999), which is consistent with prior twin studies suggesting that environmental influences may have a more pronounced impact on initiation, while genetic factors are more influential in progression to heavier use and substance-related problems (Kendler, Karkowski, Neale, & Prescott, 2000).

### Nicotine use disorder

Heritable factors contribute across the stages of cigarette smoking and NicUD, with a range of heritability estimates for nicotine dependence (ND) between ~0.30 and 0.70 (Agrawal et al., 2012; Sullivan & Kendler, 1999). Variability in reported *h*^2^ results for NicUD could, at least in part, be due to the different ways in which NicUD-related problems have been assessed [e.g. Fagerström Tolerance Questionnaire (FTQ), Fagerström Test for Nicotine Dependence (FTND)] in comparison to NicUD as determined by DSM diagnostic criteria (Cohen, Myers, & Kelly, 2002; Payne, Smith, McCracken, McSherry, & Antony, 1994).

### Cannabis use disorder

Heritability estimates from twin studies of CanUD range from ~0.51 to 0.59, slightly higher than the estimates for cannabis use/initiation (~0.40–0.48; Agrawal & Lynskey, 2006; Verweij et al., 2010). Twin and family studies have found shared genetic and environmental influences across the stages of cannabis use and abuse (Agrawal, Neale, Jacobson, Prescott, & Kendler, 2005; Van den Bree, Johnson, Neale, & Pickens, 1998). Gillespie, Neale, and Kendler (2009) explored this further, finding that *availability* of cannabis explained nearly all of the shared environmental variance in cannabis initiation and abuse, initiation mediated the influence of availability on abuse, and a large proportion of the genetic variance in abuse (62%) was shared with initiation.

### Opioid use disorder

Twin and family studies have estimated that ~50% of the liability to opioid dependence is due to additive genetic factors (Berrettini, 2017; Kendler, Jacobson, Prescott, & Neale, 2003; Tsuang, Bar, Harley, & Lyons, 2001). Tsuang et al. (1998) estimated that 38% of the variation in opioid addiction was due to genetic factors *specific* to opioids (i.e. not shared with other substances).

### Cocaine use disorder

Estimates of the heritability of CocUD range from ~0.40 to 0.80, with evidence of a common genetic vulnerability with other SUDs, especially cannabis, and little evidence of cocaine-specific genetic influences (Kendler et al., 2007).

### Genetic correlations amongst SUDs

Twin studies have also been used to assess the genetic correlations (*r*_g_) amongst substance use and SUDs. Population-based twin estimates of *r*_g_ may be less prone to biases inherent in modern genome-wide studies that rely on large biobanks with unrepresentative sample characteristics (e.g. the relatively healthy and high SES makeup of the UK Biobank). Briefly, findings from twin and family studies suggest common genetic factors shared amongst substance use (*r*_g_ = 0.14–0.31 for alcohol, tobacco, and cannabis), with stronger estimates of shared genetic overlap amongst measures of *problem* use (*r*_g_ = 0.56–0.62; Young, Rhee, Stallings, Corley, & Hewitt, 2006).

## Genetics of SUDs

Genome-wide association studies (GWAS) of SUDs have rapidly increased in both sample size and locus identification during the past 5 years (reviewed in Hancock, Markunas, Bierut, & Johnson, 2018; Johnson, Chang, & Agrawal, 2020*a*). Still, the sample sizes for SUD GWAS have lagged behind those of lifetime ever-use or consumption [e.g. drinks per week (DPW)], largely due to the additional burden associated with administering and undergoing a comprehensive SUD assessment in comparison to more readily administered survey and screener questionnaires. In addition, there are a variety of considerations in the recruitment of individuals with SUDs (Fisher & Jaber, 2019), including that these individuals may be more difficult to reach and/or less willing to participate in research studies. Sample diversity has also been limited; the majority of SUD GWAS to date are primarily composed of individuals of European ancestry and findings may not generalize to those of other ancestries.

One overarching question that has emerged from the first-generation of well-powered SUD GWAS is whether measures of non-problematic substance use have divergent genetic underpinnings from SUDs, and if so, to what extent. Another area of interest has been dissecting the genetic relationships between SUDs, other psychiatric disorders, and relevant complex traits; by leveraging large GWAS and advanced statistical genetics methods [e.g. cross-trait genetic correlations, genomicSEM (Grotzinger et al., 2019)], interesting patterns of pleiotropy have emerged (Abdellaoui, Smit, Van Den Brink, Denys, & Verweij, 2021; Hatoum et al., 2021; Jang et al., 2020). Notable SUD GWAS loci are summarized in Table 1.

**Table 1.** Summary of epidemiology, genetic epidemiology, and molecular genetic findings for substance use disorders

	Alcohol use disorder (AUD)	Nicotine use disorder (NicUD)	Cannabis use disorder (CanUD)	Opioid use disorder (OUD)	Cocaine use disorder (CocUD)^a^
SUD epidemiology and recent developments	◦ Lifetime prevalence rate = 29.1%^41^ ◦ Alcohol use and intoxication contributes to three million worldwide deaths annually^122^	◦ Lifetime prevalence rate = 27.9%^41^ ◦ Nicotine use and related-disease contributes to seven million worldwide deaths annually^123^	◦ Lifetime prevalence rate = 6.3%^41^ ◦ Recent legalization in Western countries is correlated with increased use, including among pregnant women; it remains to be seen whether this influences prevalence rates of CUD^15^	◦ Lifetime prevalence rate = 2.1%^41^ ◦ Despite lower prevalence relative to other SUDs, OUD poses large disease burden due to overdose deaths^53^	◦ Lifetime prevalence rate = 2.4%^41^ ◦ From 2012 to 2018, the rate of overdose deaths related to cocaine use increased from 1.4% to 4.5%^53^
SUD genetic epidemiology	◦ AUD heritability (*h*^2^) = 0.50–0.64^51,61,117^ ◦ SNP-heritability (*h*^2^SNP) = ~0.07–0.10^128^	◦ NicUD heritability (*h*^2^) = ~0.30–0.70^4,111^ ◦ SNP-heritability (*h*^2^SNP) = ~0.09^98^	◦ CanUD heritability (*h*^2^) = 0.40–0.80^118^ ◦ SNP-heritability (*h*^2^SNP) = ~0.07–0.12^61^	◦ OUD heritability (*h*^2^) = ~0.50^8,67,114^ ◦ SNP-heritability (*h*^2^SNP) = ~0.11^127^	◦ CocUD heritability (*h*^2^) = 0.40–0.80^69^ ◦ SNP-heritability (*h*^2^SNP) = ~0.27–0.30^14,55^
Notable GWAS risk-loci to date **BOLD** = loci have achieved *p* ≤ 5.0x10^–9^ with respective SUD in at least one study	◦ ***ADH1B***^b,c^ (rs1229984) ^17,32,36,78,84,105,121,124,128^ ◦ ***ALDH2***^b,c^ (rs671)^37,62,81,99^ ◦ ***DRD2***^b^ (e.g. rs4936277)^78,105,128^ ◦ ***KLB***^b,c^ (e.g. rs13129401) ^78,105,128^ ◦ ***GCKR***^b,c^ (rs1260326)^17,78,84,105,128^ ◦ ***SLC39A8***^b,c^ (rs13107325)^78,84,105^	◦ ***CHRNA5***^b,c^ (rs16969968)^31,45,46,84,98^ ◦ ***CHRNA5-A3-B4***^b,c^ (multiple loci)^31,45,46,84,98^ ◦ ***CHRNA4***^b,c^ (rs151176846)^45,46,84,98^ ◦ *DNMT3B*^b^ (rs910083)^46^ ◦ *DBH*^b,c^ (rs13284520)^84,98^	◦ ***FOXP2***^b^ (rs7783012)^61^ ◦ ***CHRNA2***^b^ (rs4732724)^23,61^ ◦ *EPHX2*^b^ (rs4732724)^61^ ◦ *CSMD1*^b^ (rs77378271)^107^ ◦ *PDE4B*^b^ (gene-wise)^61^	◦ ***OPRM1***^b^ (rs1799971)^127^ ◦ ***CNIH3***^b^ (rs10799590)^92^ ◦ ***KCNG2***^b^ (rs62103177)^33^ ◦ *RGMA*^b^ (rs12442183)^16^ ◦ *BEND4*^c^ (gene-wise)^96^	◦ *FAM53B*^b^ (rs2629540)^34^ ◦ *HIST1H2BD*^b^ (gene-wise)^14^ ◦ *C1QL2*^b^ (gene-wise)^55^ ◦ *STK38*^b^ (gene-wise)^55^ ◦ *KCTD20*^b^ (gene-wise)^55^
Notable genetic correlations (*r*_g_) with psychiatric/substance use traits	◦ Drinks per week (*r*_g_ = +0.77)^128^ ◦ Ever smoked regularly (*r*_g_ = +0.55)^128^ ◦ Lifetime cannabis use (*r*_g_ = +0.39) ^128^ ◦ Major depression (*r*_g_ = +0.39)^128^ ◦ Risk-taking (*r*_g_ = +0.30)^128^	◦ Alcohol dependence (*r*_g_ = +0.56)^98^ ◦ Cigarettes per day (*r*_g_ = +0.95)^98^ ◦ Major depression (*r*_g_ = +0.38)^98^ ◦ Schizophrenia (*r*_g_ = +0.16)^98^ ◦ Smoking initiation (*r*_g_ = +0.40)^98^	◦ Alcohol use disorder (*r*_g_ = +0.55)^61^ ◦ Educational attainment (*r*_g_ = −0.39)^61^ ◦ Lifetime cannabis use (*r*_g_ = +0.50)^61^ ◦ Schizophrenia (*r*_g_ = +0.31)^61^ ◦ Smoking initiation (*r*_g_ = +0.66)^61^	◦ ADHD (*r*_g_ = +0.36)^127^ ◦ Alcohol dependence (*r*_g_ = +0.73)^127^ ◦ Drinks per week (*r*_g_ = +0.38)^127^ ◦ Ever smoked regularly (*r*_g_ = +0.51)^127^ ◦ Major depression (*r*_g_ = +0.35)^127^	◦ ADHD (*r*_g_ = +0.50)^14^ ◦ Ever smoked regularly (*r*_g_ = +0.34)^14^ ◦ Major depression (*r*_g_ = +0.40)^14^ ◦ Risk-taking (*r*_g_ = +0.35)^14^ ◦ Schizophrenia (*r*_g_ = +0.20)^14^
Notable CNV and exome/genome sequencing efforts	◦ Genome-wide meta-analysis of CNV associations in AUD cases^112^: ◦ identified nine CNV regions suggestively associated with AUD (e.g. 5q21.3 deletion)	◦ Exome-chip meta-analysis fine mapped rare coding variants for nicotine use outcomes^10^: ◦ identified 124 significant associations. ◦ 1.0–2.2% of phenotypic variance explained by rare variation	◦ Low-coverage WGS found two gene regions significantly associated with CanUD^39^: ◦ C1orf110 gene (protein-coding region) ◦ *MEF2B* gene (regulatory region)	◦ Three significantly associated CNVs^82^: ◦ a 18q12.3 deletion ◦ a Xq28 deletion ◦ a 18q12.3 deletion	◦ Several targeted sequencing^44^ and CNV studies (e.g. *NSF* gene)^13^ have been conducted, although no strong evidence has emerged. CocUD whole-genome, whole-exome, and CNV studies are needed
Notable efforts incorporating non-European populations	◦ Kranzler et al. (2019)^78^: AUD cases: EUR = 34 658; AFR = 17 267; LAT = 3449; EAA = 164; SAA = 44 ◦ Walters et al. (2018)^121^: AUD cases: EUR = 11 569; AFR = 3335	◦ Quach et al. (2020)^98^: *N* = 46 213 EUR smokers; *N* = 11 787 AFR smokers ◦ Hancock et al. (2018*a*)^46^: *N* = 28 677 EUR smokers; *N* = 9925 AFR smokers	◦ Johnson et al. (2020*b*)^61^: CanUD cases: EUR = 17 068; AFR = 3848 ◦ Sherva et al. (2016)^107^: CanUD cases: EUR = 2884; AFR = 1572	◦ Zhou et al. (2020*a*, *b*)^127^: OUD: EUR cases = 8259, EUR opioid-exposed controls = 71 200; AFR cases = 4032, AFR opioid-exposed controls = 26 029	◦ Huggett and Stallings (2020*a*, *b*)^55^: CocUD: EUR cases = 3370; AFR cases = 2349 ◦ Gelernter et al. (2014*a*)^34^: provided CocUD case–control data for Huggett and Stallings (2020*a*, *b*) analyses

Abbreviations for notable samples incorporating non-European populations: European Ancestry (EUR), African Ancestry (AFR), Latino or Hispanic Ancestry (LAT); East Asian American (EAA); South Asian American (SAA).

*Note*: numeric superscripts correspond to numbered in-text citations (See Supplementary Material).

a At the time of review, CocUD sample sizes remain substantially smaller than other SUDs; thus, current CocUD findings and downstream analyses (e.g. *h*^2^SNP, *r*_g_) should be interpreted with caution and require replication in well-powered samples. Efforts to extend CocUD sample sizes are underway.

b Denotes findings with SUD or problematic use.

c Denotes findings with substance consumption measure.

### Alcohol use disorder

Up until the past 5 years, there was limited progress in the identification of replicable genetic loci associated with AUD, excepting the well-established influence of genes encoding alcohol metabolism enzymes [e.g. alcohol dehydrogenase 1B (*ADH1B*), aldehyde dehydrogenase 2 (*ALDH2*); reviewed in Edenberg & Mcclintick, 2018]. Recent studies have demonstrated that, similar to other complex traits, larger sample sizes have aided the successful detection of loci influencing AUD and alcohol-related outcomes (reviewed in Deak, Miller, & Gizer, 2019; Sanchez-Roige, Palmer, & Clarke, 2020). These have replicated genome-wide significant (GWS) associations for loci in the *ADH1B* gene (e.g. rs1229984 and rs2066702) with AUD (Gelernter et al., 2014*a*; Kranzler et al., 2019; Walters et al., 2018; Zhou et al., 2020*a*, *b*) and with various measures of alcohol use and consumption (Clarke et al., 2017; Gelernter et al., 2019; Kranzler et al., 2019; Liu et al., 2019; Sanchez-Roige et al., 2019*a*, *b*; Xu et al., 2015). Similar success has been found for loci mapped to *ALDH2* (i.e. rs671) with robust associations found with alcohol dependence and alcohol-related traits (i.e. maximum drinks, flushing response) in East-Asian and Thai populations (Gelernter et al., 2018; Li, Zhao, & Gelernter, 2012; Quillen et al., 2014), and for alcohol drinking status in East-Asian populations (Jorgenson et al., 2017).

Associations with other loci have begun to be consistently identified as well. Recent GWAS have robustly identified associations between genetic variation in *DRD2* (Dopaminergic Receptor D2) and AUD (rs4936277, rs61902812; Kranzler et al., 2019) and problematic alcohol use (PAU; rs138084129, rs6589386; Zhou et al. 2020*a*, *b*), as well as gene-based associations with alcohol problems, as indexed by Alcohol Use Disorders Identification Test (AUDIT) scores (Sanchez-Roige et al., 2019*a*, *b*). Other loci, including the *GCKR* gene (Glucokinase Regulator; lead SNP: rs1260326), are associated with AUD and alcohol use problems (Kranzler et al., 2019; Sanchez-Roige et al., 2019*a*, *b*; Zhou et al., 2020*a*, *b*), as well as consumption (Clarke et al., 2017; Kranzler et al., 2019; Liu et al., 2019; Sanchez-Roige et al., 2019*a*, *b*). A *KLB* (Klotho Beta) variant was also found to be associated with PAU (rs13129401; Zhou et al., 2020*a*, *b*), and with AUDIT measures of alcohol problems (AUDIT-P; Sanchez-Roige et al., 2019*a*, *b*) and consumption (AUDIT-C; Kranzler et al., 2019; Sanchez-Roige et al., 2019*a*, *b*). There also have been associations between variants in the *SLC39A8* gene and AUD (rs13107325; Kranzler et al., 2019), AUDIT-P (rs13135092; Sanchez-Roige et al., 2019*a*, *b*) and AUDIT-C [rs13107325 (Kranzler et al., 2019); rs13135092 (Sanchez-Roige et al., 2019*a*, *b*)].

Methodological advancements (e.g. LD score regression, Bulik-Sullivan et al., 2015) modeling aggregate genetic risk across the genome have furthered our understanding of the genetic architecture of AUD. LD score regression approaches have generated SNP-heritability (*h*^2^_SNP_) estimates of ~0.07–0.10 for measures of PAU (Zhou et al., 2020*a*, *b*), and demonstrated positive genetic correlations between PAU and smoking cigarettes regularly, lifetime cannabis use, major depression, and risk-taking (Table 1; Zhou et al., 2020*a*, *b*). Additionally, varying patterns of genetic correlations suggest only a partial genetic overlap between AUD and alcohol consumption (Sanchez-Roige et al., 2020), likely due at least in part to differences in levels of drinking and overarching pathology across samples. A recent study reported a stronger correlation between DPW and PAU (*r*_g_ = 0.77; Zhou et al., 2020*a*, *b*), highlighting that high consumption is a necessary component of AUD; however, similar to other measures of alcohol consumption (e.g. AUDIT-C), DPW has demonstrated negligible genetic correlations (*r*_g_ = −0.02 to 0.08) with other psychiatric disorders [attention-deficit hyperactivity disorder (ADHD), bipolar disorder, major depression, schizophrenia; Jang et al., 2020], while PAU has shown stronger genetic overlap with these disorders (*r*_g_ = 0.32–0.39; Zhou et al., 2020*a*, *b*). These most recent results from Jang et al. (2020) and Zhou et al. (2020*a*, *b*), respectively, suggest that while DPW is a component of problematic use, PAU seems to capture shared genetic risk with other psychiatric disorders, while DPW does not.

Despite divergent patterns of genetic overlap suggesting non-uniform genetic influences, it should be noted that genes influencing alcohol-metabolizing enzymes (e.g. *ADH1B*, *ALDH2*) directly affect alcohol consumption, and in turn, play a role in the risk of AUD development. The coding variants in these genes provide a protective effect for AUD by producing aversive effects when drinking alcohol, often resulting in lower levels of consumption and AUD risk (Edenberg & Mcclintick, 2018). However, it is likely that thousands of additional genetic loci play a role beyond the genes encoding alcohol metabolizing enzymes. Additional studies have examined subdomains of alcohol consumption, suggesting potential etiological differences between alcohol consumption *frequency* and alcohol consumption *quantity* (Mallard et al., 2020; Marees et al., 2020*b*). Specifically, alcohol consumption *quantity* was found to be more genetically similar to AUD and psychopathology, while *frequency* demonstrated negative relationships with AUD and other psychiatric outcomes, and was found to be influenced by measures of SES (Mallard et al., 2020; Marees et al., 2020*b*). Thus, evidence of genetic dissimilarity between consumption and AUD may be being driven by *frequency* of drinking, which in turn, is being influenced by indices of SES. Further studies probing this relationship will be needed to fully disentangle the nuance of the shared and unique genetic etiology across the spectrum of alcohol consumption levels (e.g. normative consumption, binge drinking) and AUD.

The largest genome-wide meta-analysis of copy number variation (CNV) and AUD to date found nine CNV regions (six deletions and three duplications) that were suggestively associated with AUD status in a sample of 3243 cases (Sulovari, Liu, Zhu, & Li, 2018). The most significant association (albeit modest: *p* = 3.8 × 10^−4^) was a deletion located on 5q21.3, a region that has previously been reported to be associated in a linkage study examining alcohol craving in a Native American population (Ehlers & Wilhelmsen, 2005).

### Nicotine use disorder

Large-scale GWAS of ND have consistently reported GWS associations with cholinergic nicotinic receptor genes. For example, one GWAS reported GWS associations between ND, as assessed by FTND scores, and the well-replicated signal found for genetic variation within the *CHRNA5-CHRNA3-CHRNB4* locus on chromosome 15 (Table 1; Hancock et al., 2018*b*). Hancock et al. (2018*b*) also reported a novel association with an intronic variant (rs910083) in the DNA methyltransferase gene (*DNMT3B*) located on chromosome 20 that was further found to be associated with heavy smoking in the UK Biobank and implicated in the development of lung cancer. A more recently expanded GWAS of ND from the Nicotine Dependence GenOmics Consortium (iNDiGO; Quach et al., 2020) provided further evidence for a top variant association in *CHRNA5* on chromosome 15 (rs16969968), and a significantly associated variant (rs151176846) in *CHRNA4* on chromosome 20 (*CHRNA4*) that was associated with ND in an earlier GWAS (Hancock et al., 2015). The iNDiGO Consortium estimated an *h*^2^_SNP_ of ~0.09 for ND (Quach et al., 2020).

There have also been large-scale efforts examining genetic contributions for other nicotine-related phenotypes (Furberg et al., 2010; Liu et al., 2019). For example, GSCAN (GWAS & Sequencing Consortium of Alcohol and Nicotine use; Liu et al., 2019) reported 467 GWS associations across a variety of smoking-related phenotypes [initiation of regular smoking, quantity of cigarettes per day (CPD), smoking cessation, and age of regular smoking initiation]. In GSCAN, the top single variant association reported for smoking outcomes was between the CPD phenotype and rs16969968 located within *CHRNA5* (Liu et al., 2019), similar to findings reported in other GWAS of smoking behaviors (Furberg et al., 2010) and ND (Hancock et al., 2015, 2018*a*; Quach et al., 2020).

Varying patterns of genetic relationships between NicUD and other smoking phenotypes have been observed. For example, smoking initiation was found to be modestly genetically correlated with ND (*r*_g_ = 0.40), while CPD and ND were highly genetically correlated (*r*_g_ = 0.95); this suggests that smoking initiation is less genetically similar to problematic nicotine use relative to CPD (Quach et al., 2020; Table 1). Genetic overlap between CPD (Liu et al., 2019) and ND (Quach et al., 2020) may be being driven, in part, by the fact that iNDiGO ND was assessed using FTND scores, a measure generally accepted as a reasonable assessment for ND but that also includes assessment for the number of cigarettes smoked per day.

There have been efforts examining rare variant associations with nicotine use. A recent exome-chip meta-analysis of 16 studies fine-mapped 124 GWS rare coding variant associations across nicotine use outcomes [i.e. CPD, pack-years (i.e. quantity of cigarette packs smoked in lifetime), smoking initiation, age of smoking initiation; Brazel et al., 2019]. Rare variation accounted for 1.0–2.2% of phenotypic variance across these traits (Brazel et al., 2019).

### Cannabis use disorder

There have been fewer replicable genome-wide discoveries for CanUD, due to small sample sizes. To date, the largest GWAS of CanUD (*N*_cases_ = 20 916), which combined data from iPSYCH, deCODE Genetics, and the PGC (see Box 1), estimated the SNP-heritability (*h*^2^_SNP_) to be ~0.12 (using an estimated prevalence of 8.5%) and identified two GWS loci: one located on chromosome 7, near the *FOXP2* gene (lead SNP: rs7783012), and the second located on chromosome 8, with brain eQTLs for *CHRNA2* and *EPHX2* (lead SNP: rs4732724; Table 1; Johnson et al., 2020*b*). *FOXP2* plays a role in synaptic plasticity and has been implicated in speech and language development, and the lead risk variant at this locus, rs7783012, has been previously associated with externalizing behaviors. The *CHRNA2* gene, which encodes the *α*-2 subunit of the neuronal nicotinic acetylcholine receptor, has been previously implicated in GWAS of CanUD (Demontis et al., 2019) as well as tobacco smoking and schizophrenia, both of which are phenotypically and genetically correlated with CanUD (Table 1). The *EPHX2* gene may be involved in the metabolism of cannabinoids, making this an attractive candidate gene for CanUD, but it is currently unclear whether *EPHX2* or *CHRNA2* may be mechanistically responsible for driving the association between this locus and CanUD. Another notable finding is a GWS variant (rs77378271) in the *CSMD1* gene, which has previously been linked to schizophrenia; Sherva et al. identified this variant in their European-ancestries GWAS of DSM-IV cannabis dependence (*N* = 8754) as well as the trans-ancestral meta-analysis (*N* = 14 754) that included individuals of both European- and African-ancestries (Sherva et al., 2016). However, this gene (as is the case for most CanUD risk variants proposed so far) has not been replicated in other GWAS of CanUD.

Box 1.

**Table:** 

Glossary of relevant terms:
**Copy number variation (CNV):** A type of structural genetic variation impacting a region of DNA, resulting in the deletion or duplication of genetic information. **Endophenotypes:** Any measurable component between genotype and a trait of interest. Useful for examining subfacets and/or transdiagnostic features of SUDs. **Epigenome-wide Association Study (EWAS):** Study examining associations between a phenotype and epigenetic markers (e.g. levels of DNA methylation). **Expression quantitative trait loci (eQTL):** Genetic loci influencing expression levels of mRNA (messenger RNA) in disease-relevant tissues (e.g. brain eQTLs). **Genome-wide Association Study (GWAS):** A genetic study testing associations between a phenotype and genetic variants (measured or imputed) across the genome. **Genotype-Tissue Expression (GTEx):** Publicly-available database allowing for the query of a specific genetic variant's involvement in tissue-specific gene expression. **Heritability (*h*2)**: The proportion of variation in a phenotype due to genetic factors; traditionally measured using pedigree information (i.e. twin- or family-based studies), but can be assessed using molecular genetic approaches. This is a population-level measure that can vary across time and environments. **LD-score regression:** Method that requires only GWAS summary statistics to estimate SNP-based heritability and genetic correlations between traits of interest. **Linkage disequilibrium (LD):** The phenomenon wherein nearby genetic variants are inherited non-independently of each other; if two variants are in linkage equilibrium, they are inherited independently of each other (i.e. not correlated or linked). **Multi-omics (‘omics):** The study of multiple levels of biological information (e.g. epigenome, transcriptome) in a systems-based approach. **Pharmacogenomics:** Studies examining how an individual's genetic variation impacts their pharmacological response to medications. **Polygenic score (PGS):** An aggregate score of an individual's genetic predisposition for a certain trait, calculated using a ‘discovery’ GWAS of that phenotype. **Rare variation:** Genetic variation occurring at lower frequencies in a population, generally defined as having a minor allele frequency (MAF) <1%. **Single nucleotide polymorphism (SNP):** A single base-pair change in the DNA that is relatively common (MAF >1–5%) in the population. **SNP-based heritability (*h*2SNP):** The proportion of variation in a phenotype (i.e. heritability) that is accounted for by measured molecular genetic information (i.e. GWAS data). Estimate of the additive genetic variance that can be explained by common SNPs.
Glossary of relevant large-scale genetic efforts for substance use and SUDs:
deCODE**:** A large, population-based series of studies based out of Reykjavik, Iceland examining genetic influences on complex traits (e.g. SUDs). FinnGen**:** A national research initiative leveraging genetic and digital information from Finland national registries to inform biomedicine and personalized healthcare. **GSCAN (GWAS & Sequencing Consortium of Alcohol and Nicotine use):** An international genetic consortium aimed toward conducting large-scale meta-analyses of GWAS, low-frequency non-synonymous variation, and whole-genome sequencing studies for alcohol and nicotine use traits. **iNDiGO (Nicotine Dependence GenOmics Consortium):** A large-scale genomic consortium examining genetic influences of nicotine dependence and nicotine-related traits across diverse ancestry groups. **iPSYCH (Initiative for Integrative Psychiatric Research):** A large national project founded in Denmark in 2012 funded by the Lundbeck Foundation examining genetic and environmental influences for mental health disorders (e.g. SUDs) in over 130 000 Danish individuals. **MVP (Million Veteran Program):** One of the world's largest biobanks including genetic, environmental, and medical information from United States Military Veterans. **PGC (Psychiatric Genomics Consortium):** A large collaborative effort spanning 800+ investigators, 36 countries, and >400 000 subjects aimed toward elucidating the genetic contributions across psychiatric disorders. The PGC consists of 14 working groups, including the Substance Use Disorders (PGC-SUD) Working Group. **UK Biobank:** A large-scale biomedical database and research resource consisting of genetic and health information from greater than 500 000 UK participants.

Mirroring findings from twin and family studies, GWAS of CanUD have identified significant genetic overlap between CanUD and other SUDs and measures of substance use. CanUD showed significant positive genetic correlations with smoking initiation, ND, CPD, DPW, and AUD (*r*_g_ ranging from 0.31 to 0.66; Table 1; Johnson et al., 2020*b*).

Similar to findings for alcohol, recent GWAS of CanUD have found divergence between cannabis use and CanUD, both at the level of individual risk loci as well as genetic relationships with other traits and disorders. Despite a significant correlation of 0.50 between CanUD and lifetime cannabis use, 12 of 22 traits tested had significantly different genetic correlations with CanUD *v.* cannabis use (Johnson et al., 2020*b*). For example, lifetime cannabis ever-use shows positive genetic correlations with education and age at first birth, and a negative correlation with BMI (+, +, −; Pasman et al., 2018), while CanUD shows genetic correlations in the opposite direction of effect for these three traits (−, −, +; Johnson et al., 2020*b*). This suggests that, while necessary for the development of CanUD, cannabis initiation is at least partly genetically distinct from CanUD.

One recent study used low-pass whole-genome sequencing (WGS) to study CanUD in two samples, one Native American tribal community and one family-based sample of primarily European ancestry (Gizer, Bizon, Gilder, Ehlers, & Wilhelmsen, 2018). Two significant regions were identified in a meta-analysis of the two samples: one protein-coding region, *C1orf110*, and one regulatory region in the *MEF2B* gene.

### Opioid use disorder

To date, GWAS of OUD have identified significant loci near the *KCNG2*, *KCNC1*, *APBB2*, *CNIH3*, *RGMA*, and *OPRM1* genes (Table 1; Cheng et al., 2018; Gelernter et al., 2014*b*; Nelson et al., 2016; Polimanti et al., 2020). The largest OUD GWAS to date, conducted in a total of 114 759 individuals (15 756 cases), observed a significant functional coding variant (rs1799971) in the *OPRM1* gene (Zhou et al., 2020*a*, *b*). Other substance use traits (e.g. ever-smoked, alcohol dependence) and psychiatric disorders (e.g. ADHD, schizophrenia) were positively correlated with OUD (Table 1). Zhou et al. (2020*a*, *b*) estimated the *h*^2^_SNP_ of OUD to be 0.11 (s.e. = 0.02).

While there has been less work examining potential differences between the genetic etiology of OUD compared to lifetime ever-use of opioids or non-dependent opioid use, a study from the PGC observed some genetic differences when comparing opioid-dependent individuals, opioid-exposed controls, and opioid-unexposed controls (Polimanti et al., 2020). There were significant relationships between a risk-taking polygenic score (PGS) and the contrast of opioid dependence and unexposed controls, as well as opioid-exposed controls *v.* unexposed controls. A PGS for neuroticism was associated with opioid dependence (contrasted with both unexposed and exposed controls), but not with the unexposed *v.* exposed controls contrast, consistent with the authors' hypothesis that neuroticism is associated with negative affect being related to dependence but not simply exposure (Polimanti et al., 2020). These results suggest that the definition of controls in SUD GWAS needs to be carefully considered.

The largest CNV study of OUD to date identified three common CNVs (two deletions and one duplication) significantly associated with OUD, and several rare CNVs with large effect sizes that reached suggestive levels of evidence (Li et al., 2015). Interestingly, the three significant CNVs (a 18q12.3 deletion, a Xq28 deletion, and a 1q21.3 duplication) were associated with OUD in both the African-ancestries sample (*N*_cases_ = 547) and a combined, trans-ancestral meta-analysis with the European-ancestries samples (*N*_total−cases_ = 1601).

### Cocaine use disorder

CocUD GWAS sample sizes have lagged behind those of licit substances. Thus far, there has been one GWS variant identified: rs2629540, located in the *FAM53B* gene (Gelernter et al., 2014*a*). Huggett and Stallings (2020*a*, *b*) applied a gene-wise test to these data and identified four significant genes: *C1QL2*, *STK38*, and *KCTD20* in European Americans (*N* = 3176), and *NDUFB9* in African Americans (*N* = 3370). A meta-analysis of CocUD (all European-ancestry; *N*_cases_ = 2085, *N*_controls_ = 4293) identified an association with *HIST1H2BD* in a gene-based test (Cabana-Domínguez, Shivalikanjli, Fernàndez-Castillo, & Cormand, 2019). They also found positive genetic correlations with schizophrenia, ADHD, major depression, and risk-taking, in line with phenotypic correlations (despite the number of cases being less than recommended for LD score regression; Table 1). Another recent study used cluster analyses to identify CocUD subtypes with reduced phenotypic heterogeneity, one potential barrier to identifying significant genetic variants for psychiatric disorders (Sun, Kranzler, Gelernter, & Bi, 2020). Still, few genetic findings have replicated amongst GWAS of CocUD; we expect the number of robust, replicable findings to increase with larger sample sizes (similar to other SUDs).

The estimated *h*^2^_SNP_ of CocUD is larger than for other SUDs; Huggett and Stallings (2020*a*) estimated *h*^2^_SNP_ = 0.28 (s.e. = 0.14) in their genome-wide analysis of CocUD, while Cabana-Dominguez estimated *h*^2^_SNP_ = 0.27–0.30 (s.e. = 0.03–0.06), depending on the estimation method (LDSC *v.* GCTA-GREML; Cabana-Domínguez et al., 2019). In contrast, the *h*^2^_SNP_ for the other SUDs discussed here range from 0.07 to 0.12, depending on the phenotype and prevalence (Table 1). The estimations for CocUD heritability may be inaccurate due to under-powered sample sizes – it will be interesting to see if this pattern of larger *h*^2^_SNP_ is borne out in future large-scale GWAS of CocUD.

## Clinical and therapeutic implications

Recent efforts have been made to bring molecular genetic findings from large-scale GWAS of SUDs to translational relevance, especially in terms of genetic prediction of SUDs. However, the complexity of SUDs makes genetic prediction efforts difficult and potentially fraught – they are polygenic, heterogeneous, and multifactorial disorders heavily influenced by environmental factors (including access).

PGS have shown promise for the stratification of individuals at risk by their polygenic ‘load’ for some health conditions; for example, one successful non-SUD application of PGS was reported for coronary disease, where individuals in the highest quintile of genetic risk had an approximately 90% increase in relative risk of experiencing an adverse coronary event compared to individuals in the lowest quintile of genetic risk (Khera et al., 2016). Current SUD PGS explain a relatively small proportion of variance (generally 1–5%) in SUD-related outcomes, especially relative to other known risk factors (SES, SUD family history, comorbid psychiatric disorders; Barr et al., 2020). This limits their current clinical utility. Furthermore, the best-powered GWAS of SUDs to date have been conducted primarily in samples of European ancestries, limiting their predictive utility to individuals who are also of European ancestry (Martin, Daly, Robinson, Hyman, & Neale, 2019). Finally, PGS can be difficult to interpret in layperson's terms (i.e. being in the 95th percentile of polygenic risk for alcohol dependence does not mean you have a 95% chance of developing the disorder). Further research is needed to fully understand the potential benefits, and possible harms, of incorporating genetic information (e.g. PGS) into SUD treatment planning (Driver, Kuo, & Dick, 2020; Lebowitz, 2019; Lebowitz & Ahn, 2018).

Other efforts to utilize molecular genetics for precision medicine purposes have included pharmacogenetic studies, i.e. identifying genetic variability in pharmaceutical treatment response and efficacy. Some GWAS have, post-hoc, identified gene targets for treatment. For example, several genes identified in GWAS of smoking behaviors, including *CHRNA7*, *CHRNA5*, *CHRNA4*, and *CHRNB2*, have been found to moderate the effect of Varenicline, a smoking cessation treatment that operates as a partial agonist at the nicotine acetylcholine a2b4 receptor (King et al., 2012). However, case-only GWAS comparing treatment responders to non-responders are more likely to uncover pharmacogenetic variability than GWAS of the disorder itself. To date, many pharmacogenomic studies of SUDs have been candidate gene-focused (e.g. dopaminergic pathway genes, Patriquin, Bauer, Soares, Graham, & Nielsen, 2015) and have had limited success. However, a recent opioid dosing GWAS identified a variant close to the *OPRM1* locus affecting methadone dosing requirements in African-ancestry individuals (Smith et al., 2017). Another recent GWAS of AUD treatment outcomes identified multiple loci associated with medication-specific outcomes and provided evidence of polygenic contributions to AUD treatment response (Biernacka et al., 2021). The increasing availability of large, longitudinal datasets with access to electronic health records and genotype data may enable more systematic, unbiased investigations into the interactions between genetic variation and medication efficacy (Hartwell & Kranzler, 2019).

Drug repurposing, or identifying a new indication for an existing therapeutic, has emerged as another promising way to bring GWAS findings to therapeutic relevance (So et al., 2017). Bupropion is a classic example of a drug repurposed to some success: while it was originally used as a treatment for depression, clinicians discovered that it aided in smoking cessation (Fava, 2018). In a proof of principle study, So et al. (2017) suggested several repositioning candidates for psychiatric disorders by connecting imputed transcriptomic profiles from GWAS data to drug-induced gene expression profiles, but this has not yet been done for SUDs. While there have not been any success stories to date for repurposed drugs for SUDs discovered using GWAS data, this is an intriguing path forward, particularly for SUDs, where there are still typically few effective pharmaceutical treatments available.

At this time, expectations for the use of complex genetics in clinical and therapeutic settings should be tempered; genome-wide data of SUDs are not informative enough to improve upon factors already assessed in the clinic for diagnostic and risk prediction purposes, but there remains the potential for pharmacogenomic and drug repositioning efforts to make an impact on the treatment of SUDs in the future.

## Conclusions and future priorities

Molecular genetic studies of SUDs have undergone massive advances during the past 5 years. Increased GWAS sample sizes and the incorporation of additional ‘omics data’ have contributed to a better understanding of the molecular mechanisms and biological pathways underlying SUDs (Kapoor et al., 2019). In our conclusions, we highlight possible next steps and suggested priorities for the field of SUD genetics:

### Increased diversity of SUD GWAS

The majority of GWAS of SUDs to date are composed primarily of individuals of European-ancestry, and thus, the generalizability of these findings to other ancestry groups is uncertain. This gap has the potential to further exacerbate health disparities for individuals of diverse ancestry. This raises the need for efforts to study SUDs in transancestral populations, such as the All of Us Research Program. As shown in Table 1, GWAS of SUDs have included relatively more diverse samples compared to other psychiatric disorders, but the numbers of non-European samples are still well below the European-ancestry sample sizes.

Linkage-disequilibrium patterns differ across populations, which is one reason that discovery GWAS of European ancestry may not lead to maximally-predictive PGS in non-European ancestry target samples (Martin et al., 2019). It is thus imperative, in the interest of scientific discovery and ensuring that everyone benefits equally from those discoveries, that future SUD GWAS focus on increasing the number of samples of non-European ancestry.

### Integration of functional genomic data and cross-species translational models

Recent studies have begun to leverage multi-omics data to identify genes and biological processes associated with SUDs. For example, Kapoor et al. (2019) performed differential gene expression analysis on prefrontal cortex tissue from 65 AUD cases and 73 controls, identifying relevant genes and molecular pathways including upregulation of pathways related to immune responses. Markunas et al. (2020) conducted the first epigenome-wide association study (EWAS) of smoking in human post-mortem brain tissue (specifically the nucleus accumbens); they identified seven DNA methylation (DNAm) biomarkers, three of which were located near genes previously implicated as blood-based DNAm biomarkers of smoking and four of which were novel (*ABLIM3*, *APCDD1L*, *MTMR6*, and *CTCF*). Another recent study (Marees et al., 2020*a*) used GTEx (Genotype-Tissue Expression; Ardlie et al., 2015) data to assess the role of eQTLs in six substance use traits; using this approach, they identified novel loci not identified in the original GWAS for five of the traits. Despite demonstrating progress, these studies also highlight current limitations for SUDs, especially the lack of SUD-specific and cell-type-specific multi-omics data sources. For example, while GTEx (Ardlie et al., 2015) is a valuable resource for general tissue-specific gene expression patterns, the data provide no information about substance-induced transcriptomic changes. There is a need for more SUD-specific tissue samples. Evidence from Kapoor et al. (2019) and the Markunas et al. (2020) EWAS further highlight the importance of examining both brain and other tissues (e.g. blood, liver) in substance-specific studies: drugs can have peripheral effects, but brain-specific biomarkers may provide greater insight into the neurobiological effects of substance exposure.

Another potential direction is the integration of human genetic data with findings from animal models of addiction endophenotypes (Reynolds et al., 2020). The substance use genetics literature is rich with rodent models of addictive behaviors (e.g. positive reinforcement via self-administration paradigms, withdrawal avoidance and drug-seeking). Despite the challenges that must be overcome to integrate human and animal genetic data (e.g. handling non-orthologous genes), rodent endophenotypes may provide insight into the neurobiological mechanisms linking genes to SUD risk. Another issue is that there is no certain way to cross-map animal and human phenotypes, limiting the opportunities for translation. However, as a recent proof of principle, one study (Huggett, Bubier, Chesler, & Palmer, 2020*c*) found modest but significant overlap in differentially expressed genes and gene networks when comparing human CocUD case–control data with mice in a cocaine *v.* saline solution self-administration paradigm, suggesting commonalities in the reward circuitry of human CocUD and self-administration paradigms in rodents.

### Refinement of phenotypes and ascertainment strategies

A key priority for future genetics studies of SUDs is further examining the implications of broad *v.* deep phenotyping approaches and different sample-ascertainment strategies. Prior studies (reviewed in Sanchez-Roige et al., 2020; Sanchez-Roige & Palmer, 2020) suggest that consumption measures (e.g. alcohol intake frequency, cannabis initiation) have divergent patterns of genetic correlation relative to their respective SUDs. However, contrasts of substance use and use disorder are complicated by several issues, including the recall period: while most measures of SUDs are lifetime diagnoses, measures of substance use are often assessed within a recent timeframe (e.g. past year). Additionally, large, unrepresentative samples (e.g. the UK Biobank is skewed toward older individuals with high SES, the Million Veteran Program is skewed heavily toward males) can lead to collider bias (Munafò, Tilling, Taylor, Evans, & Davey Smith, 2018), biases can arise from misreporting and longitudinal changes (Xue et al., 2021), and there is a complicated interplay between genetic and sociological factors in the context of substance use and the development of SUDs (see larger discussion in previous AUD genetics section; Marees et al., 2020*b*). These issues complicate efforts to examine distinctions between the genetics of substance use and SUDs. As mentioned earlier for alcohol, there are instances in which the genetics of ‘use’ are intimately linked with the genetics of use disorder (e.g. *ADH1B* and *ALDH2* variants exerting their effects via decreased likelihood of alcohol consumption). Furthermore, there is evidence that both substance use (e.g. lifetime cannabis use, ever smoked cigarettes regularly) and SUDs (e.g. CanUD) are strongly related to general externalizing behaviors (Linnér et al., 2020). Still, a recent preprint identified a common genetic factor that underlies SUDs but is *not* shared with measures of substance use, nor other psychiatric disorders (Hatoum et al., 2021), suggesting that SUDs are not simply the combination of substance exposure and psychopathology. Collectively, these findings suggest the importance of assessing a variety of measures of substance use and SUDs (via clinical diagnoses or shorter questionnaires) from multiple types of samples to further elucidate the genetic architecture of consumption measures compared to problematic use across SUDs.

Another promising direction forward in terms of ascertainment strategy is the development of population-based biobanks with embedded family designs (e.g. FinnGen; https://www.finngen.fi/en). Even in the absence of molecular genetic data, national registries have previously been used in innovative ways to examine the influence of genes and family environment on SUD outcomes in offspring (e.g. the triparental design explored in Kendler, Ohlsson, Sundquist, & Sundquist, 2015). One benefit of genotyped population-based datasets with embedded families is that researchers can apply new genetic methods that leverage relatedness patterns to better understand the ways in which parents may influence children's substance use trajectories both ‘directly’ (passing on SUD risk alleles) and ‘indirectly’ (through family environment) (Kong et al., 2018).

Finally, there are multiple substance classes not covered in this review, including hallucinogens, ‘club drugs’, and inhalants. These substance classes have been included in a handful of twin and family studies examining drug use, but no well-powered GWAS exist. Future GWAS efforts will be informative for how the genetics of these additional SUDs overlap with or diverge from well-studied SUDs.

### Conclusion

Recent years have brought substantial progress in advancing our understanding of the genetic architecture of SUDs and other substance use behaviors (e.g. consumption quantity), and relating these findings to etiologically-relevant processes for the development of SUDs. The field will continue to see significant advances in genetic discovery as larger sample sizes of individuals of diverse ancestry begin to become realized. It is the hope that these continued advancements will have clinically meaningful implications for SUD prevention and treatment in the future.

## Note

1 Please see Box 1 for glossary of key terms used in the present review.

## Supplementary material

For supplementary material accompanying this paper visit https://doi.org/10.1017/S0033291721000969. click here to view supplementary material
