# Genetics of substance use disorders: a review – CORRIGENDUM

**DOI:** 10.1017/S0033291722000629

**Published:** 2022-03

**Authors:** Joseph D. Deak, Emma C. Johnson

**Affiliations:** 1 Department of Psychiatry, Yale School of Medicine, New Haven, CT, USA; 2 Department of Psychiatry, Veterans Affairs Connecticut Healthcare Center, West Haven, CT, USA; 3 Department of Psychiatry, Washington University School of Medicine, St. Louis, MO, USA

This article was published in Psychological Medicine with some errors in Table 1. The AFR and EUR case numbers have been swapped and the OUD counts have been corrected to read: [EUR cases = 10544; EUR controls = 72613] and [AFR cases = 5212; AFR controls = 26876]. A reference was also not cited appropriately in the main text and has been fixed.

The authors apologise for this error.
