# Supplementary material for: Genetics of substance use disorders: a review
Source: Psychol Med. 2021 Apr 21;51(13):2189–200. doi: 10.1017/S0033291721000969 (PMC8477224; doi:10.1017/S0033291721000969)
Supplement: Supplementary file 1 [file S0033291721000969sup001.docx]

Supplemental Material for Genetics of substance use disorders: A review

Numbered References

1. Abdellaoui, A., Smit, D. J. A., Van Den Brink, W., Denys, D., & Verweij, K. J. H. (2021). Genomic relationships across psychiatric disorders including substance use disorders. Drug and Alcohol Dependence, 220, 108535.https://doi.org/10.1016/j.drugalcdep.2021.108535
2. Agrawal, A., & Lynskey, M. T. (2006). The genetic epidemiology of cannabis use, abuse and dependence. Addiction, 101(6), 801–812.
3. Agrawal, A., Neale, M., Jacobson, K., Prescott, C. A. & Kendler, K. S. (2005) Illicit drug use and abuse/dependence: modeling of two-stage variables using the CCC approach. Addictive Behavior, 30, 1043–1048.
4. Agrawal, A., Verweij, K. J. H., Gillespie, N. A., Heath, A. C., Lessov-Schlaggar, C. N., Martin, N. G., … Lynskey, M. T. (2012). The genetics of addiction—A translational perspective. Translational Psychiatry, 2(7), e140–e140.
5. American Psychiatric Association: Diagnostic and Statistical Manual of Mental Disorders: Diagnostic and Statistical Manual of Mental Disorders, Fifth Edition (2013). Arlington, VA: American Psychiatric Association.
6. Ardlie KG, Deluca DS, Segre AV, Sullivan TJ, Young TR, Gelfand ET, … Dermitzakis ET (2015). The Genotype-Tissue Expression (GTEx) pilot analysis: Multitissue gene regulation in humans. Science 348, 648–660.
7. Barr PB, Ksinan A, Su J, Johnson EC, Meyers JL, Wetherill L, … Dick DM (2020). Using polygenic scores for identifying individuals at increased risk of substance use disorders in clinical and population samples. Translational Psychiatry, 10, 196.
8. Berrettini, W. (2017). A brief review of the genetics and pharmacogenetics of opioid use disorders. Dialogues in Clinical Neuroscience, 19(3), 229.
9. Biernacka, J. M., Coombes, B. J., Batzler, A., Geske, J. R., Ho, A. M., Frank, J., … Karpyak, V. M.. (2021). Genetic Contributions to Alcohol Use Disorder Treatment Outcomes: A Genome-wide Pharmacogenomics Study. https://doi.org/10.1101/2021.02.03.21251107
10. Brazel, D. M., Jiang, Y., Hughey, J. M., Turcot, V., Zhan, X., Gong, J., … Surendran, P. (2019). Exome chip meta-analysis fine maps causal variants and elucidates the genetic architecture of rare coding variants in smoking and alcohol use. Biological Psychiatry, 85(11), 946–955.
11. Brick, L. A., Micalizzi, L., Knopik, V. S., & Palmer, R. H. (2019). Characterization of DSM-IV opioid dependence among individuals of European ancestry. Journal of Studies on Alcohol and Drugs, 80(3), 319–330.
12. Bulik-Sullivan BK, Loh P-R, Finucane HK, Ripke S, Yang J, Patterson N, … Neale BM (2015). LD Score regression distinguishes confounding from polygenicity in genome-wide association studies. Nature Genetics Nature Genetics 47, 291–295.
13. Cabana-Domínguez, J., Roncero, C., Grau-López, L., Rodríguez-Cintas, L., Barral, C., Abad, A. C., … Arenas, C. (2016). A highly polymorphic copy number variant in the NSF gene is associated with cocaine dependence. Scientific Reports, 6(1), 1–10.
14. Cabana-Domínguez, J., Shivalikanjli, A., Fernàndez-Castillo, N., & Cormand, B. (2019). Genome-wide association meta-analysis of cocaine dependence: Shared genetics with comorbid conditions. Progress in Neuro-Psychopharmacology and Biological Psychiatry, 94, 109667.
15. Cerdá M, Wall M, Keyes KM, Galea S, & Hasin D (2012). Medical marijuana laws in 50 states: Investigating the relationship between state legalization of medical marijuana and marijuana use, abuse and dependence. Drug and Alcohol Dependence Drug and Alcohol Dependence 120, 22–27.
16. Cheng Z, Zhou H, Sherva R, Farrer LA, Kranzler HR, & Gelernter J (2018). Genome-wide Association Study Identifies a Regulatory Variant of RGMA Associated With Opioid Dependence in European Americans. Biological Psychiatry Biological Psychiatry 84, 762–770.
17. Clarke, T.-K., Adams, M. J., Davies, G., Howard, D. M., Hall, L. S., Padmanabhan, S., … Hayward, C. (2017). Genome-wide association study of alcohol consumption and genetic overlap with other health-related traits in UK Biobank (N= 112 117). Molecular Psychiatry, 22(10), 1376–1384.
18. Cohen, L.M., Myers, M.G. & Kelly, J.F. (2002). Assessment of Nicotine Dependence Among Substance Abusing Adolescent Smokers: A Comparison of the DSM-IV Criteria and the Modified Fagerström Tolerance Questionnaire. Journal of Psychopathology and Behavioral Assessment 24, 225–233. https://doi.org/10.1023/A:1020722915204.
19. Compton, W. M., Dawson, D. A., Goldstein, R. B., & Grant, B. F. (2013). Crosswalk between DSM-IV dependence and DSM-5 substance use disorders for opioids, cannabis, cocaine and alcohol. Drug and Alcohol Dependence, 132(1–2), 387–390.
20. Cotto KC, Wagner AH, Feng Y-Y, Kiwala S, Coffman AC, Spies G, … Griffith M (2018). DGIdb 3.0: a redesign and expansion of the drug–gene interaction database. Nucleic Acids Research Nucleic Acids Research 46, D1068–D1073.
21. Crist, R. C., Reiner, B. C., & Berrettini, W. H. (2019). A review of opioid addiction genetics. Current Opinion in Psychology, 27, 31–35.
22. Deak, J. D., Miller, A. P., & Gizer, I. R. (2019). Genetics of alcohol use disorder: A review. Current Opinion in Psychology, 27, 56–61.
23. Degenhardt, L., & Hall, W. (2012). Extent of illicit drug use and dependence, and their contribution to the global burden of disease. The Lancet, 379(9810), 55–70.
24. Demontis, D., Rajagopal, V. M., Thorgeirsson, T. E., Als, T. D., Grove, J., Leppälä, K., … Reginsson, G. W. (2019). Genome-wide association study implicates CHRNA2 in cannabis use disorder. Nature Neuroscience, 22(7), 1066.
25. Driver, M. N., Kuo, S. I.-C., & Dick, D. M. (2020). Genetic feedback for psychiatric conditions: Where are we now and where are we going. American Journal of Medical Genetics Part B: Neuropsychiatric Genetics.
26. Edenberg HJ, & Mcclintick JN (2018). Alcohol Dehydrogenases, Aldehyde Dehydrogenases, and Alcohol Use Disorders: A Critical Review. Alcoholism Clinical and Experimental Research Alcoholism Clinical and Experimental Research 42, 2281–2297.
27. Ehlers, C. L., & Wilhelmsen, K. C. (2005). Genomic scan for alcohol craving in Mission Indians. Psychiatric Genetics, 15(1), 71–75.
28. Fava M (2018). The promise and challenges of drug repurposing in psychiatry. World Psychiatry World Psychiatry 17, 28–29.
29. Fisher, C. B., & Jaber, R.. (2019). Ethical Issues in Substance-Use Prevention Research. In Advances in Prevention Science (pp. 281–299). Advances in Prevention Science. https://doi.org/10.1007/978-3-030-00627-3_18
30. Forgetta V, Jiang L, Vulpescu NA, Hogan MS, Chen S, Morris JA, … Richards JB (2020). An Effector Index to Predict Causal Genes at GWAS Loci. bioRxiv 2020.06.28.171561; doi: https://doi.org/10.1101/2020.06.28.171561.
31. Furberg, H., Kim, Y., Dackor, J., Boerwinkle, E., Franceschini, N., Ardissino, D., … Merlini, P. A. (2010). Genome-wide meta-analyses identify multiple loci associated with smoking behavior. Nature Genetics, 42(5), 441.
32. Gelernter, J., Kranzler, H. R., Sherva, R., Almasy, L., Koesterer, R., Smith, A. H., … Farrer, L. A. (2014a). Genome-wide association study of alcohol dependence: significant findings in African- and European-Americans including novel risk loci. Molecular psychiatry, 19(1), 41–49. https://doi.org/10.1038/mp.2013.145
33. Gelernter, J., Kranzler, H. R., Sherva, R., Koesterer, R., Almasy, L., Zhao, H., & Farrer, L. A. (2014b). Genome-wide association study of opioid dependence: Multiple associations mapped to calcium and potassium pathways. Biological Psychiatry, 76(1), 66–74.
34. Gelernter, J., Sherva, R., Koesterer, R., Almasy, L., Zhao, H., Kranzler, H. R., & Farrer, L. (2014c). Genome-wide association study of cocaine dependence and related traits: FAM53B identified as a risk gene. Molecular Psychiatry, 19(6), 717–723.
35. Gelernter, J., Sherva, R., Zhao, H., Kranzler, H., & Farrer, L. (2019). Initial Results From An Opioid Dependence Whole Exome Sequencing Study. European Neuropsychopharmacology, 29, S732.
36. Gelernter, J., Sun, N., Polimanti, R., Pietrzak, R. H., Levey, D. F., Lu, Q., … Aslan, M. (2019). Genome-wide association study of maximum habitual alcohol intake in > 140,000 US European and African American veterans yields novel risk loci. Biological Psychiatry, 86(5), 365–376.
37. Gelernter J, Zhou H, Nuñez YZ, Mutirangura A, Malison RT, & Kalayasiri R (2018). Genomewide association study of alcohol dependence and related traits in a Thai population. Alcoholism Clinical and Experimental Research.
38. Gillespie, N.A., Neale, M.C., & Kendler, K.S. (2009), Pathways to cannabis abuse: a multi‐stage model from cannabis availability, cannabis initiation and progression to abuse. Addiction, 104: 430-438. doi:10.1111/j.1360-0443.2008.02456.x
39. Gizer, I. R., Bizon, C., Gilder, D. A., Ehlers, C. L., & Wilhelmsen, K. C. (2018). Whole genome sequence study of cannabis dependence in two independent cohorts. Addiction Biology, 23(1), 461–473.
40. Goldman, D., Oroszi, G., & Ducci, F. (2005). The genetics of addictions: Uncovering the genes. Nature Reviews Genetics, 6(7), 521–532. https://doi.org/10.1038/nrg1635
41. Grant, B. F., Chou, S. P., Saha, T. D., Pickering, R. P., Kerridge, B. T., Ruan, W. J., … Fan, A. (2017). Prevalence of 12-month alcohol use, high-risk drinking, and DSM-IV alcohol use disorder in the United States, 2001-2002 to 2012-2013: Results from the National Epidemiologic Survey on Alcohol and Related Conditions. JAMA Psychiatry, 74(9), 911–923.
42. Grant, B. F., Saha, T. D., Ruan, W. J., Goldstein, R. B., Chou, S. P., Jung, J., … Hasin, D. S. (2016). Epidemiology of DSM-5 Drug Use Disorder: Results From the National Epidemiologic Survey on Alcohol and Related Conditions–III. JAMA Psychiatry, 73(1), 39–47. https://doi.org/10.1001/jamapsychiatry.2015.2132
43. Grotzinger, A. D., Rhemtulla, M., De Vlaming, R., Ritchie, S. J., Mallard, T. T., Hill, W. D., … Tucker-Drob, E. M. (2019). Genomic structural equation modelling provides insights into the multivariate genetic architecture of complex traits. Nature Human Behaviour, 3(5), 513–525. https://doi.org/10.1038/s41562-019-0566-x
44. Haller, G., Kapoor, M., Budde, J., Xuei, X., Edenberg, H., Nurnberger, J., … Almasy, L. (2014). Rare missense variants in CHRNB3 and CHRNA3 are associated with risk of alcohol and cocaine dependence. Human Molecular Genetics, 23(3), 810–819.
45. Hancock, D. B., Reginsson, G. W., Gaddis, N. C., Chen, X., Saccone, N. L., Lutz, S. M., … Zink, F. (2015). Genome-wide meta-analysis reveals common splice site acceptor variant in CHRNA4 associated with nicotine dependence. Translational Psychiatry, 5(10), e651–e651.
46. Hancock DB, Guo Y, Reginsson GW, Gaddis NC, Lutz SM, Sherva R, … Johnson EO (2018). Genome-wide association study across European and African American ancestries identifies a SNP in DNMT3B contributing to nicotine dependence. . Molecular Psychiatry Molecular Psychiatry 23, 1911–1919.
47. Hancock, D.B., Markunas, C. A., Bierut, L. J., & Johnson, E. O. (2018). Human genetics of addiction: New insights and future directions. Current Psychiatry Reports, 20(2), 8.
48. Hartwell EE & Kranzler HR (2019). Pharmacogenetics of alcohol use disorder treatments: an update, Expert Opinion on Drug Metabolism & Toxicology, 15:7, 553-564, DOI: 10.1080/17425255.2019.1628218
49. Hasin, D. S., O’Brien, C. P., Auriacombe, M., Borges, G., Bucholz, K., Budney, A., … Grant, B. F. (2013). DSM-5 Criteria for Substance Use Disorders: Recommendations and Rationale. American Journal of Psychiatry, 170(8), 834–851. https://doi.org/10.1176/appi.ajp.2013.12060782
50. Hatoum, A. S., Johnson, E. C., Polimanti, R., Zhou, H., Walters, R., Gelernter, J., … Agrawal, A. (2021). The Addiction Genetic Factor a (g): A Unitary Genetic Vulnerability Characterizes Substance Use Disorders and Their Associations with Common Correlates. medRxiv 2021.01.26.21250498.
51. Heath A.C., Bucholz K.K., Madden P.A., Dinwiddie S.H., Slutske W.S., Bierut L.J., … Martin N.G. (1997). Genetic and environmental contributions to alcohol dependence risk in a national twin sample: consistency of findings in women and men. Psychological Medicine, 27(6), 1381–1396.
52. Heatherton, T. F., Kozlowski, L. T., Frecker, R. C., & Fagerström, K.-O. (1991). The Fagerström test for nicotine dependence: A revision of the Fagerstrom Tolerance Questionnaire. British Journal of Addiction, 86(9), 1119–1127.
53. Hedegaard M, Miniño A, & Warner M (2020). NCHS Data Brief: No. 356: January 2020: Drug overdose deaths in the United States, 1999–2018. Retrieved from https://stacks.cdc.gov/view/cdc/84647-h.pdf.
54. Hu Y, Lu Q, Powles R, Yao X, Yang C, Fang F, … Zhao H (2017). Leveraging functional annotations in genetic risk prediction for human complex diseases. PLoS Computational Biology PLoS Computational Biology 13, e1005589.
55. Huggett, S. B., & Stallings, M. C. (2020a). Cocaine’omics: Genome-wide and transcriptome-wide analyses provide biological insight into cocaine use and dependence. Addiction Biology, 25(2), e12719.
56. Huggett, S. B., & Stallings, M. C. (2020b). Genetic Architecture and Molecular Neuropathology of Human Cocaine Addiction. Journal of Neuroscience, 40 (27) 5300-5313.
57. Huggett SB, Bubier JA, Chesler EJ, & Palmer RHC (2020c). Do Meso‐Limbic Gene Expression Findings from Mouse Models of Cocaine Self‐Administration Recapitulate Human Cocaine Use Disorder?. Genes, Brain and Behavior 20:e12689. https://doi.org/10.1111/gbb.12689
58. Jang, S.-K., Saunders, G., Liu, M., Jiang, Y., Liu, D. J., Vrieze, S., & Team, 23andMe Research. (2020). Genetic correlation, pleiotropy, and causal associations between substance use and psychiatric disorder. Psychological Medicine, 1–11.
59. Jensen, K. P. (2016). A review of genome-wide association studies of stimulant and opioid use disorders. Molecular Neuropsychiatry, 2(1), 37–45.
60. Johnson, E. C., Chang, Y., & Agrawal, A. (2020a). An Update on the Role of Common Genetic Variation Underlying Substance Use Disorders. Current Genetic Medicine Reports, 1–12.
61. Johnson E.C., Demontis D, Thorgeirsson T.E., Walters R.K., Polimanti R., Hatoum A.S., … Agrawal, A. (2020b). A large-scale genome-wide association study meta-analysis of cannabis use disorder. The Lancet Psychiatry. 7: 1032–45
62. Jorgenson E, Thai KK, Hoffmann TJ, Sakoda LC, Kvale MN, Banda Y, … Choquet H (2017). Genetic contributors to variation in alcohol consumption vary by race/ethnicity in a large multi-ethnic genome-wide association study. Molecular Psychiatry 22, 1359–1367.
63. Kapoor M, Wang J-C, Farris SP, Liu Y, Mcclintick J, Gupta I, … Goate A (2019). Analysis of whole genome-transcriptomic organization in brain to identify genes associated with alcoholism. Translational Psychiatry.
64. Kendler KS (2001). Twin Studies of Psychiatric Illness. Archives of General Psychiatry Archives of General Psychiatry 58, 1005.
65. Kendler, K. S., Ohlsson, H., Karriker-Jaffe, K. J., Sundquist, J., & Sundquist, K. (2017). Social and economic consequences of alcohol use disorder: A longitudinal cohort and co-relative analysis. Psychological Medicine, 47(5), 925.
66. Kendler, K.S., Chen, X., Dick, D., Maes, H., Gillespie, N., Neale, M. C., & Riley, B. (2012). Recent advances in the genetic epidemiology and molecular genetics of substance use disorders. Nature Neuroscience, 15(2), 181.
67. Kendler, K.S., Jacobson, K. C., Prescott, C. A., & Neale, M. C. (2003). Specificity of genetic and environmental risk factors for use and abuse/dependence of cannabis, cocaine, hallucinogens, sedatives, stimulants, and opiates in male twins. American Journal of Psychiatry, 160(4), 687–695.
68. Kendler, K.S., Karkowski, L. M., Neale, M. C., & Prescott, C. A. (2000). Illicit psychoactive substance use, heavy use, abuse, and dependence in a US population-based sample of male twins. Archives of General Psychiatry, 57(3), 261–269.
69. Kendler, K.S., Myers, J., & Prescott, C. A. (2007). Specificity of genetic and environmental risk factors for symptoms of cannabis, cocaine, alcohol, caffeine, and nicotine dependence. Archives of General Psychiatry, 64(11), 1313–1320.
70. Kendler, K. S., Ohlsson, H., Sundquist, J., & Sundquist, K. (2015). Triparental Families: A New Genetic-Epidemiological Design Applied to Drug Abuse, Alcohol Use Disorders, and Criminal Behavior in a Swedish National Sample. American Journal of Psychiatry, 172(6), 553–560. https://doi.org/10.1176/appi.ajp.2014.14091127
71. Kendler, K.S., Prescott, C. A., Myers, J., & Neale, M. C. (2003). The structure of genetic and environmental risk factors for common psychiatric and substance use disorders in men and women. Archives of General Psychiatry, 60(9), 929–937.
72. Kessler RC (2004). The epidemiology of dual diagnosis. Biological Psychiatry 56, 730–737.
73. Khera, A. V., Emdin, C. A., Drake, I., Natarajan, P., Bick, A. G., Cook, N. R., … Kathiresan, S.. (2016). Genetic Risk, Adherence to a Healthy Lifestyle, and Coronary Disease. New England Journal of Medicine, 375(24), 2349–2358. http://doi.org/10.1056/nejmoa1605086
74. King DP, Paciga S, Pickering E, Benowitz NL, Bierut LJ, Conti DV, … Park PW (2012). Smoking Cessation Pharmacogenetics: Analysis of Varenicline and Bupropion in Placebo-Controlled Clinical Trials. Neuropsychopharmacology 37, 641–650.
75. Kong, A., Thorleifsson, G., Frigge, M. L., Vilhjalmsson, B. J., Young, A. I., Thorgeirsson, T. E., … Stefansson, K. (2018). The nature of nurture: Effects of parental genotypes. Science, 359(6374), 424–428. https://doi.org/10.1126/science.aan6877
76. Koob GF & Le Moal M (2001). Drug Addiction, Dysregulation of Reward, and Allostasis. Neuropsychopharmacology 24, 97–129.
77. Koopmans, J. R., Slutske, W. S., Van Baal, G. C. M., & Boomsma, D. I. (1999). The influence of religion on alcohol use initiation: Evidence for genotype X environment interaction. Behavior Genetics, 29(6), 445–453.
78. Kranzler, H. R., Zhou, H., Kember, R. L., Smith, R. V., Justice, A. C., Damrauer, S., … Reid, J. (2019). Genome-wide association study of alcohol consumption and use disorder in 274,424 individuals from multiple populations. Nature Communications, 10(1), 1–11.
79. Lebowitz, M. S. (2019). The Implications of Genetic and Other Biological Explanations for Thinking about Mental Disorders. Hastings Center Report, 49, S82–S87.
80. Lebowitz, M. S., & Ahn, W. (2018). Blue genes? Understanding and mitigating negative consequences of personalized information about genetic risk for depression. Journal of Genetic Counseling, 27(1), 204–216.
81. Li D, Zhao H, & Gelernter J (2012). Strong protective effect of the aldehyde dehydrogenase gene (ALDH2) 504lys (*2) allele against alcoholism and alcohol-induced medical diseases in Asians. Human Genetics 131, 725–737.
82. Li, D., Zhao, H., Kranzler, H. R., Li, M. D., Jensen, K. P., Zayats, T., … Gelernter, J. (2015). Genome-wide association study of copy number variations (CNVs) with opioid dependence. Neuropsychopharmacology, 40(4), 1016–1026.
83. Linnér, R. K., Mallard, T. T., Barr, P. B., Sanchez-Roige, S., Madole, J. W., Driver, M. N., … Dick, D. M. (2020). Multivariate genomic analysis of 1.5 million people identifies genes related to addiction, antisocial behavior, and health. BioRxiv. https://doi.org/10.1101/2020.10.16.342501
84. Liu, M., Jiang, Y., Wedow, R., Li, Y., Brazel, D. M., Chen, F., …Vrieze, S. (2019). Association studies of up to 1.2 million individuals yield new insights into the genetic etiology of tobacco and alcohol use. Nature Genetics, 51(2), 237–244.
85. Mallard TT, Savage JE, Johnson EC, Huang Y, Edwards AC, Hottenga JJ, … Sanchez-Roige S (2020). Multivariate GWAS elucidates the genetic architecture of alcohol consumption and misuse, corrects biases, and reveals novel associations with disease. bioRxiv 2020.09.21.304196; doi: https://doi.org/10.1101/2020.09.21.304196
86. Marees, A. T., Gamazon, E. R., Gerring, Z., Vorspan, F., Fingal, J., van den Brink, W., … Sherva, R. (2020a). Post-GWAS analysis of six substance use traits improves the identification and functional interpretation of genetic risk loci. Drug and Alcohol Dependence, 206, 107703.
87. Marees, A. T., Smit, D. J. A., Ong, J.-S., Macgregor, S., An, J., Denys, D., … Derks, E. M. (2020b). Potential influence of socioeconomic status on genetic correlations between alcohol consumption measures and mental health. Psychological Medicine, 50(3), 484–498. https://doi.org/10.1017/s0033291719000357
88. Markunas CA, Semick SA, Quach BC, Tao R, Deep-Soboslay A, Carnes MU, … Hancock DB (2020). Genome-wide DNA methylation differences in nucleus accumbens of smokers vs. nonsmokers. Neuropsychopharmacology 46, 554–560
89. Martin AR, Daly MJ, Robinson EB, Hyman SE, & Neale BM (2019). Predicting Polygenic Risk of Psychiatric Disorders. Biological Psychiatry 86, 97–109.
90. Munafò, M. R., Tilling, K., Taylor, A. E., Evans, D. M., & Davey Smith, G. (2018). Collider scope: when selection bias can substantially influence observed associations. International Journal of Epidemiology, 47(1), 226–235. https://doi.org/10.1093/ije/dyx206
91. National Institute of Drug Abuse. Overdose death rates. 2018. Available at: https://www.drugabuse.gov/related‐topics/trends‐statistics/overdose‐death‐rates. Accessed September 09, 2020.
92. Nelson EC, Agrawal A, Heath AC, Bogdan R, Sherva R, Zhang B, … Montgomery GW (2016). Evidence of CNIH3 involvement in opioid dependence. Molecular Psychiatry 21, 608–614.
93. Patriquin, M. A., Bauer, I. E., Soares, J. C., Graham, D. P., & Nielsen, D. A. (2015). Addiction pharmacogenetics: a systematic review of the genetic variation of the dopaminergic system. Psychiatric genetics, 25(5), 181–193. https://doi.org/10.1097/YPG.0000000000000095
94. Pasman, J. A., Verweij, K. J., Gerring, Z., Stringer, S., Sanchez-Roige, S., Treur, J. L., … Ong, J.-S. (2018). GWAS of lifetime cannabis use reveals new risk loci, genetic overlap with psychiatric traits, and a causal effect of schizophrenia liability. Nature Neuroscience, 21(9), 1161–1170.
95. Payne, T. J., Smith, P. O., McCracken, L. M., McSherry, W. C., & Antony, M. M. (1994). Assessing nicotine dependence: A comparison of the fagerström tolerance questionnaire (FTQ) with the fagerström test for nicotine dependence (FTND) in a clinical sample. Addictive Behaviors, 19, 307–317.10.1016/0306-4603(94)90032-9.
96. Polimanti, R., Walters, R. K., Johnson, E. C., McClintick, J. N., Adkins, A. E., Adkins, D. E., ... Gelernter, J. (2020). Leveraging genome-wide data to investigate differences between opioid use vs. Opioid dependence in 41,176 individuals from the Psychiatric Genomics Consortium. Molecular Psychiatry, 1–15.
97. Prom-Wormley, E. C., Ebejer, J., Dick, D. M., & Bowers, M. S. (2017). The genetic epidemiology of substance use disorder: A review. Drug and Alcohol Dependence, 180, 241–259. https://doi.org/10.1016/j.drugalcdep.2017.06.040
98. Quach, B. C., Bray, M. J., Gaddis, N. C., Liu, M., Palviainen, T., Minica, C. C., … Hancock, D. B. (2020). Expanding the genetic architecture of nicotine dependence and its shared genetics with multiple traits. *Nature Communications*, *11*(1). https://doi.org/10.1038/s41467-020-19265-z
99. Quillen EE, Chen X-D, Almasy L, Yang F, He H, Li X, … Gelernter J (2014). ALDH2 is associated to alcohol dependence and is the major genetic determinant of “daily maximum drinks” in a GWAS study of an isolated rural chinese sample. American Journal of Medical Genetics Part B Neuropsychiatric Genetics 165, 103–110.
100. Reynolds T, Johnson EC, Huggett SB, Bubier JA, Palmer RHC, Agrawal A, … Chesler EJ (2020). Interpretation of psychiatric genome-wide association studies with multispecies heterogeneous functional genomic data integration. Neuropsychopharmacology 46, 86–97
101. Salvatore, J. E., Savage, J. E., Barr, P., Wolen, A. R., Aliev, F., Vuoksimaa, E., … Kaprio, J. (2018). Incorporating Functional Genomic Information to Enhance Polygenic Signal and Identify Variants Involved in Gene-by-Environment Interaction for Young Adult Alcohol Problems. Alcoholism: Clinical and Experimental Research, 42(2), 413–423.
102. Sanchez-Roige, S., Fontanillas, P., Elson, S. L., Team, 23andMe Research, Gray, J. C., de Wit, H., … Palmer, A. A. (2019). Genome-wide association study of alcohol use disorder identification test (AUDIT) scores in 20 328 research participants of European ancestry. Addiction Biology, 24(1), 121–131.
103. Sanchez-Roige, S., & Palmer, A. A. (2020). Emerging phenotyping strategies will advance our understanding of psychiatric genetics. Nature Neuroscience, 1–6.
104. Sanchez-Roige, S., Palmer, A. A., & Clarke, T.-K. (2020). Recent efforts to dissect the genetic basis of alcohol use and abuse. Biological Psychiatry, 87(7), 609–618.
105. Sanchez-Roige, S., Palmer, A. A., Fontanillas, P., Elson, S. L., 23andMe Research Team, the S. U. D. W. G. of the P. G. C., … Clarke, T.-K. (2019). Genome-wide association study meta-analysis of the Alcohol Use Disorders Identification Test (AUDIT) in two population-based cohorts. American Journal of Psychiatry, 176(2), 107–118.
106. Sey NYA, Hu B, Mah W, Fauni H, Mcafee JC, Rajarajan P, … Won H (2020). A computational tool (H-MAGMA) for improved prediction of brain-disorder risk genes by incorporating brain chromatin interaction profiles. Nature Neuroscience 23, 583–593.
107. Sherva R, Wang Q, Kranzler H, Zhao H, Koesterer R, Herman A, … Gelernter J (2016). Genome-wide Association Study of Cannabis Dependence Severity, Novel Risk Variants, and Shared Genetic Risks. JAMA Psychiatry 73, 472.
108. Smith AH, Jensen KP, Li J, Nunez Y, Farrer LA, Hakonarson H, … Gelernter J (2017). Genome-wide association study of therapeutic opioid dosing identifies a novel locus upstream of OPRM1. Molecular Psychiatry 22, 346–352.
109. So HC, Chau CK, Chiu WT, Ho KS, Lo CP, Yim SH & Sham PC (2017). Analysis of genome-wide association data highlights candidates for drug repositioning in psychiatry. Nature Neuroscience 20, 1342–1349.
110. Stone, A. L., Becker, L. G., Huber, A. M., & Catalano, R. F. (2012). Review of risk and protective factors of substance use and problem use in emerging adulthood. Addictive Behaviors, 37(7), 747–775.
111. Sullivan, P., & Kendler, K. (1999). The genetic epidemiology of smoking. Nicotine & Tobacco Research, 1(1), 51–57. https://doi.org/10.1080/14622299050011811
112. Sulovari, A., Liu, Z., Zhu, Z., & Li, D. (2018). Genome-wide meta-analysis of copy number variations with alcohol dependence. The Pharmacogenomics Journal, 18(3), 398–405. https://doi.org/10.1038/tpj.2017.35
113. Sun, J., Kranzler, H. R., Gelernter, J., & Bi, J. (2020). A genome-wide association study of cocaine use disorder accounting for phenotypic heterogeneity and gene–environment interaction. Journal of Psychiatry & Neuroscience: JPN, 45(1), 34.
114. Tsuang, M. T., Bar, J. L., Harley, R. M., & Lyons, M. J. (2001). The Harvard twin study of substance abuse: What we have learned. Harvard Review of Psychiatry, 9(6), 267–279.
115. Tsuang MT, Lyons MJ, Meyer JM, Doyle T, Eisen SA, Goldberg J, … Eaves L (1998). Co-occurrence of Abuse of Different Drugs in Men. Archives of General Psychiatry 55, 967.
116. Van den Bree, M. B., Johnson, E. O., Neale, M. C., & Pickens, R. W. (1998). Genetic and environmental influences on drug use and abuse/dependence in male and female twins. Drug and Alcohol Dependence, 52(3), 231–241.
117. Verhulst, B., Neale, M. C., & Kendler, K. S. (2015). The heritability of alcohol use disorders: A meta-analysis of twin and adoption studies. Psychological Medicine, 45(5), 1061.
118. Verweij KJH, Zietsch BP, Lynskey MT, Medland SE, Neale MC, Martin NG, … Vink JM (2010). Genetic and environmental influences on cannabis use initiation and problematic use: a meta-analysis of twin studies. Addiction 105, 417–430.
119. Viken, R. J., Kaprio, J., Koskenvuo, M., & Rose, R. J. (1999). Longitudinal analyses of the determinants of drinking and of drinking to intoxication in adolescent twins. Behavior Genetics, 29(6), 455–461.
120. Wainschtein, P., Jain, D. P., Yengo, L., Zheng, Z., Cupples, L. A., Shadyab, A. H., … Psaty, B. M. (2019). Recovery of trait heritability from whole genome sequence data. BioRxiv, 588020. https://doi.org/10.1101/588020
121. Walters, R. K., Polimanti, R., Johnson, E. C., McClintick, J. N., Adams, M. J., Adkins, A. E., … Agrawal, A. (2018). Transancestral GWAS of alcohol dependence reveals common genetic underpinnings with psychiatric disorders. Nature Neuroscience, 21(12), 1656–1669. https://doi.org/10.1038/s41593-018-0275-1
122. World Health Organization, 2018. Global Status Report on Alcohol and Health 2018 Ed, World Health Organization, Geneva, Switzerland, 2018.
123. World Health Organization. WHO Report on the Global Tobacco Epidemic, 2017: Monitoring Tobacco Use and Prevention Policies. World Health Organization; 2017.
124. Xu, K., Kranzler, H. R., Sherva, R., Sartor, C. E., Almasy, L., Koesterer, R., … Gelernter, J. (2015). Genomewide Association Study for Maximum Number of Alcoholic Drinks in European Americans and African Americans. Alcoholism, clinical and experimental research, 39(7), 1137–1147. https://doi.org/10.1111/acer.12751
125. Xue, A., Jiang, L., Zhu, Z., Wray, N. R., Visscher, P. M., Zeng, J., & Yang, J. (2021). Genome-wide analyses of behavioural traits are subject to bias by misreports and longitudinal changes. Nature Communications 12, 20211. https://doi.org/10.1038/s41467-020-20237-6
126. Young, S. E., Rhee, S. H., Stallings, M. C., Corley, R. P., & Hewitt, J. K. (2006). Genetic and Environmental Vulnerabilities Underlying Adolescent Substance Use and Problem Use: General or Specific?. Behavior Genetics, 36(4), 603–615. https://doi.org/10.1007/s10519-006-9066-7
127. Zhou H, Rentsch CT, Cheng Z, Kember RL, Nunez YZ, Sherva RM, … Gelernter J (2020). Association of OPRM1 Functional Coding Variant With Opioid Use Disorder. JAMA Psychiatry 77(10):1072-1080
128. Zhou, H., Sealock, J. M., Sanchez-Roige, S., Clarke, T.-K., Levey, D. F., Cheng, Z., … Gelernter, J. (2020). Genome-wide meta-analysis of problematic alcohol use in 435,563 individuals yields insights into biology and relationships with other traits. Nature Neuroscience, 1–10.
